# Phosphorylation of the phytosulfokine peptide receptor PSKR1 controls receptor activity

**DOI:** 10.1093/jxb/erx030

**Published:** 2017-02-23

**Authors:** Christine Kaufmann, Michael Motzkus, Margret Sauter

**Affiliations:** 1 Entwicklungsbiologie und Physiologie der Pflanzen, Christian-Albrechts-Universität Kiel, Am Botanischen Garten 5, 24118 Kiel, Germany

**Keywords:** Arabidopsis, calcium, calmodulin, growth regulation, peptide signaling, phytosulfokine receptor, pull-down, receptor-like kinase, receptor phosphorylation.

## Abstract

The phytosulfokine peptide receptor PSKR1 is modified by phosphorylation of its cytoplasmic kinase domain. We analyzed defined phosphorylation sites by site-directed mutagenesis with regard to kinase activity *in vitro* and receptor activity *in planta*. S696 and S698 in the juxtamembrane (JM) domain are phosphorylated *in planta*. The phosphomimetic S696D/S698D replacements resulted in reduced transphosphorylation activity of PSKR1 kinase *in vitro* but did not reduce autophosphorylation activity. Growth-promoting activity of the PSKR1(S696D/S698D) receptor isoform was impaired in the shoot but not in the root. The JM domain thus seems to be important for phosphorylation of a target protein required for shoot growth promotion. The phosphomimetic replacement T998D at the C-terminus (CT) abolished kinase activity *in vitro* but not receptor function *in planta*, indicating that additional levels of regulation exist *in planta*. A possible mode of receptor regulation is the interaction with regulatory proteins such as the calcium sensor calmodulin (CaM). We show that the previously reported binding of CaM2 to PSKR1 is calcium-dependent, occurs predominately to the hypophosphorylated soluble PSKR1 kinase, and does not significantly change PSKR1 kinase activity. In conclusion, our results show that peptide signaling of growth by PSKR1 is regulated by differential phosphorylation of the juxtamembrane and C-terminal domains of the intracellular receptor part and suggest that interaction of PSKR1 with CaM serves a function other than the regulation of kinase activity.

## Teaser

Phosphorylation of the phytosulfokine peptide receptor PSKR1 differentially affects auto- and transphosphorylation activity, binding to Ca^2+^-calmodulin, and has differential effects on shoot and root growth.

## Introduction

Plant peptides act as signaling molecules in developmental processes, growth regulation, and stress responses. They are of diverse nature with regard to synthesis, modification, and activities (Tavormina *et al.*, 2015), yet the known receptors that perceive peptide signals all belong to the class of leucine-rich repeat receptor-like kinases (LRR RLKs). The disulfated pentapeptide phytosulfokine (PSK) regulates growth and biotic interactions (Sauter, 2015). It is perceived by the LRR RLKs PSKR1 (phytosulfokine receptor 1) and PSKR2 (phytosulfokine receptor 2). PSK has been shown to bind to the island domain of PSKR1 that intersects between LRR17 and LRR18 of the extracellular receptor domain (Matsubayashi *et al.*, 2002; Wang *et al.*, 2015) from where the signal is transmitted to the intracelluar receptor kinase domain (PSKR1-KD).

Receptor kinases act via phosphorylation of downstream signaling or effector proteins. Protein kinases share a conserved basic structure yet have unique properties such as substrate specificity and interactions with other proteins. It is essential for a receptor that the kinase can be maintained at an inactive state to prevent unwanted signaling. Hence regulation of kinase activity is a hallmark of receptor kinases.

Information on kinase activity can be derived from structural analysis. However, in some cases only the kinase core is amenable for crystal structure analysis, such that information on a regulatory role of N- or C-termini cannot be obtained (Taylor *et al.*, 2012), as is the case for the brassinosteroid receptor BRI1 (brassinosteroid insensitive 1) that was crystallized as an N-terminally truncated protein (Bojar *et al.*, 2014). BRI1 belongs to the same subgroup (subgroup X) of the LRR RLK family as PSKR1, PSKR2, and PSYR1 (plant peptide-containing sulfated tyrosine 1 receptor) (Shiu and Bleecker, 2001; Matsubayashi *et al.*, 2006; Amano *et al.*, 2007). For PSKR1, the extracellular LRR region with the ligand-binding island domain, but not the intracellular receptor part, has been structurally characterized to date (Wang *et al.*, 2015).

**Fig. 1.**
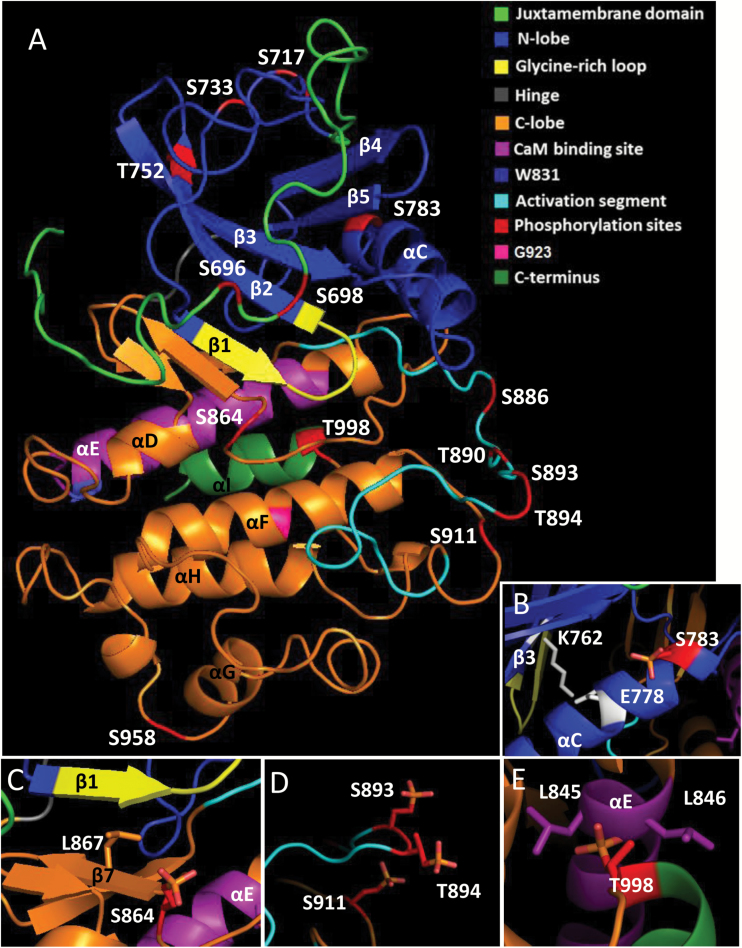
Homology model of PSKR1. (A) The structure of PSKR1-KD was modeled with Phyre2 and visualized with PyMOL version 1.2. The phosphorylation sites identified by Hartmann *et al.* (2015) are highlighted in red. (B) αC harbors the phosphosite S783 and the salt bridge-forming E778. (C) The ATP binding cleft is located between the Gly-rich loop and β7. (D) The side-chain of S911 points to the activation segment and is in close proximity to the phosphates of S893 and T894. (E) Phosphorylation of T998 interferes with αE that harbors the CaM binding site. Two leucine residues adjacent to the T998 phosphate may contribute to steric obstruction.

The intracellular PSKR1-KD is structured into a juxtamembrane (JM) domain, the kinase proper with its 12 conserved subdomains, and a short C-terminus (CT) (Fig. 1) (Hartmann *et al.*, 2015). The general features of protein kinases are readily recognized in PSKR1. In general, protein kinases consist of a small N-terminal lobe (up to amino acid I806 in PSKR1) that consists mainly of β-sheets and a larger C-terminal lobe (starting with M810 in PSKR1) predominately made up of hydrophobic α-helices (Kornev and Taylor, 2015). Sandwiched between the N- and C-lobes is the ATP binding site next to a cleft that accomodates the substrate (Taylor and Kornev, 2011).

PSKR1 belongs to the RD kinases in which the catalytic aspartate (D) in the catalytic loop in subdomain VIb is preceded by an arginine (R) (Nolen *et al.*, 2004). In RD kinases, access to the substrate binding site is controlled by the activation loop (AL) (Johnson *et al.*, 1996) (Fig. 1; for a rotating homology model of PSKR1-KD see Supplementary Video S1 available at *JXB* online). Most RD kinases are activated via phosphorylation of their AL, as also shown recently for PSKR1 (Hartmann *et al.*, 2015). For visualization of single amino acid side-chains and phospho groups of PSKR1-KD see Supplementary Fig. S1. Phosphorylation of the AL is a conserved mode of receptor kinase regulation that results in more efficient binding of substrate and/or phosphotransfer and hence in increased kinase activity (Adams, 2003). PSKR1 homo-oligomerizes *in situ* (Ladwig *et al.*, 2015) and was shown to autophosphorylate *in vitro*, indicating a self-regulatory activation mechanism (Hartmann *et al.*, 2015). The AL of PSKR1 harbors several phosphosites. Phosphomimetic and phosphoablative site-directed point mutations of these sites showed that *in vitro* activity of the PSKR1 kinase and *in planta* activity of the PSKR1 receptor are controlled at the level of AL phosphorylation (Hartmann *et al.*, 2015).

In addition, PSKR1 is phosphorylated at sites other than the AL. Phosphosites are present both in the N-lobe and the C-lobe of the kinase, and outside of the kinase proper in the JM region and at the CT (Hartmann *et al.*, 2015) (Fig. 1A and Supplementary Fig. S2). These sites have not been characterized previously. The JM domain, CT, and the C-terminal half of the C lobe with the αGαHαI helices lie outside the catalytic kinase core. They are not directly involved in substrate phosphorylation and may mediate non-catalytic functions that nonetheless influence kinase or receptor activity, such as tethering of substrates or receptor–protein interactions (Kung and Jura, 2016). To gain better insight into the regulation of PSKR1 at the level of protein modification, we analyzed the contribution of these phosphosites with respect to the regulation of kinase and receptor activity.

A known non-catalytic function of PSKR1 is its binding to the calcium sensor calmodulin (CaM) (Hartmann *et al.*, 2014). In *Arabidopsis thaliana*, all four isoforms of CaM interact with PSKR1 via an amphipathic α-helix in subdomain VIa (Fig. 1A and Supplementary Fig. S2). Mutation of a conserved tryptophane (W831) to a serine within this α-helix impairs CaM binding and PSKR1 activity *in planta*, indicating that W831 is an essential amino acid. In this study, we set out to elucidate a possible link between PSKR1-KD phosphorylation and the PSKR1-KD/CaM interaction.

## Materials and methods

### Plant materials and transformation

Plants of *Arabidopsis thaliana* (L.) Heynh., ecotype Columbia-0, were grown in 2:1 potting soil/sand mixture. To avoid contamination with insect larvae, the mixture was frozen at –80 °C for 2 d. For root growth measurements, seeds were sterilized in 2% (v/v) NaOCl solution for 15 min, washed five times with autoclaved water, and laid out on half-concentrated modified Murashige-Skoog medium (Duchefa) and 1.5% (w/v) sucrose, solidified with 0.4% (w/v) Gelrite (Duchefa) in square plates. Plates were placed for 2 d at 4 °C in the dark for stratification. Seedlings were grown under long-day conditions (16 h, 70 µM photons m^–2^ s^−1^) at 22 °C.

For plant transformation, the *pskr1-3 pskr2-1* (abbreviated as *r1 r2*) double knock-out mutant was used as background. The *r1 r2* line has been described previously (Stührwohldt *et al.*, 2011; Hartmann *et al.*, 2013). Plants were transformed with *Agrobacterium tumefaciens* GV3101 using a modified floral dip method (Clough and Bent, 1998). The *Agrobacterium* was applied twice in droplets to floral organs with 1 week in between treatments. Homozygous transgenic plants were selected by spraying with 200 µM glufosinate ammonium (Basta, AgrEvo).

Transgene expression was verified by reverse-transcription polymerase chain reaction (RT-PCR). As a control, *ACTIN2* (*ACT2*) expression was analyzed with the forward primer 5′-CAAAGACCAGCTCTTCCATCG-3′ and the reverse primer 5′-AGGTCCAGGAATCGTTCACAG-3′, resulting in a 427-bp fragment. Primers used to amplify *PSKR1* transcripts were 5′-GTTTCGGAGTTGTGCTTCTCGAG-3′ and 5′-CCAAGAGACTAACTGTTGAGTCGTTG-3′, with a product size of 251 bp. In short, 1 µg of RNA was reverse-transcribed in 20 µl, of which 1 µl was used for the amplification of *ACTIN2* cDNA and 2 µl for the amplification of *PSKR1* cDNA.

Projected rosette area was determined by the rosette tracker plugin (De Vylder, 2012) for Fiji/ImageJ open-source software (https://imagej.net/Fiji) using photographs of 4-week-old plants. Plants were photographed using an SMZ18 binocular microscope (Nikon). Root lengths were determined with the software NIS Elements 4.4.0 (Nikon). Plant height of 6-week-old plants was measured with a ruler.

### *Point mutation of* PSKR1, *heterologous protein expression, protein purification, and kinase assay*

To introduce point mutations in *PSKR1*, an overlap extension PCR (Higuchi *et al.*, 1988) was carried out with site-specific oligonucleotides (Supplementary Table S1). For the cytoplasmic kinase domain (KD), the PCR product was ligated into pETDuet-1 (Merck) using the restriction sites *Sal*I and *Avr*II, which results in an N-terminally His-tagged PSKR1 cytoplasmic domain (H_6_-PSKR1-KD). The resulting vector was transformed into *Escherichia coli* BL21 (DE3) pLys. The heterologous expression of recombinant proteins was performed in lysogeny broth (LB) Luria/Miller liquid medium with 100 µg ml^–1^ ampicillin after induction with 1 mM isopropyl-β-D-thiogalactopyranoside (IPTG) for 16 h at 20 °C. Cells were harvested, resuspended in extraction buffer (50 mM sodium phosphate, pH 8.0, 300 mM NaCl), incubated with 1 mg ml^–1^ lysozyme for 20 min on ice, disrupted in a French Press (SIM-AMINCO, Spectronic Instruments), and sonicated three times for 10 s with a SONIFIER® B-12 Cell Disruptor (Branson Sonic Power Company). Crude extract was centrifuged for 30 min at 14 000 *g* at 4 °C. H_6_-PSKR1-KD was purified with 50 µl Talon resin in a Pierce® Spin Column (Thermo Scientific) at native conditions. To that end, the supernatant was incubated with Talon resin in a rotator for 1 h at room temperature (RT). The resin was washed three times with washing buffer (50 mM sodium phosphate, pH 8.0, 300 mM NaCl, 5 mM imidazole). Affinity-purified proteins were eluted with 50 µl of elution buffer (50 mM sodium phosphate, pH 8.0, 300 mM NaCl, 150 mM imidazole) for 5 min at RT. The protein concentration was determined by measuring the absorbance at 280 nm with a NanoDrop 2000 (Thermo Scientific). The *in vitro* kinase activities were determined in 50 mM HEPES (pH 7.4), 1 mM DTT, 10 mM MgCl_2_, 10 mM MnCl_2_, 0.2 mM unlabelled ATP, 20 µCi of [γ -^32^P] ATP, 0.25 µg of affinity-purified His_6_–PSKR1-KD, and 0.5 µg of myelin basic protein (MBP) as a universal substrate for 1 h at 25 °C. Reactions were stopped by adding SDS loading buffer. Proteins were separated on a 15% polyacrylamide gel by SDS-PAGE and stained with Coomassie Brilliant Blue. Gels were subsequently dried and exposed to an X-ray film. The kinase and MBP bands were excised from the dried gels, mixed with scintillation liquid (Ultima Gold, PerkinElmer), and the radioactivity was determined (Tri-Carb 2910 TR instrument, PerkinElmer) in counts per minute (cpm). The background signal of the gel was subtracted and activities were calculated as cpm ng^–1^ kinase protein for autophosphorylation and in cpm ng^–1^ MBP for transphosphorylation activity.

To clone full-length receptor variants (FL), the Gateway™ cloning system (Life Technologies) was used. The respective point-mutated PSKR1 sequence was ligated into a modified pENTR™ 1A Dual Selection vector (Thermo Fisher Scientific) using the restricting sites *Sal*I and *Not*I, which results in a PSKR1-GFP fusion protein. The fusion construct was transferred to the overexpression vector pB7WG2.0 (Karimi *et al.*, 2002) with an LR reaction to drive *PSKR1-GFP* expression under the control of the 35S Cauliflower Mosaic Virus (CaMV) promoter.

### Cloning and expression of MaBP-FLAG-CaM2

The open reading frame of At2g41110 (*CaM2*) was amplified with the oligonucleotides 5′-TTTAAACCATGGCGG ATCAGCTCACAGAC-3′ containing an *Nco*I site and 5′-AAATTTGATATCTCACTTATCATCATCATCCTTATAAT CGACATCATCAAGCTTAGC CATCATAACCTTCACAAAC-3′ with an *Eco*RV site added. After restriction enzyme digestion, the product was ligated into the pMAL™-c5X vector (New England Biolabs, NEB) resulting in a *MaBP*-(*maltose binding protein*)*-FLAG-CaM2* fusion construct. Proper amplification was verified by sequencing prior to transformation into *E. coli* BL21(DE3) cells. Cells were grown in 30 ml LB medium, supplemented with 0.2% (w/v) glucose and 100 µg ml^–1^ ampicillin. After induction of protein expression with 0.3 mM IPTG, cells were incubated for 2 h at 37 °C, harvested, resuspended in 4 ml extraction buffer (20 mM Tris/HCl, pH 7.5, 200 mM NaCl), disrupted in a French Press, and sonicated three times for 10 s with a SONIFIER® B-12 Cell Disruptor. After centrifugation for 30 min at 21 000 *g* and 4 °C, the supernatant was collected and frozen at −80 °C until use.

### Pull-down assay

Equivalent amounts of H_6_-PSKR1-KD and H_6_-PSKR1-KD(W831S) crude extract were incubated with 20 µl MaBP-FLAG-CaM2 crude extract in 20mM Tris-HCl, pH 7.5, 200mM NaCl supplemented with 100 µM CaCl_2_ or 100 µM MgCl_2_ as indicated. For the analysis of autophosphorylated H_6_-PSKR1-KD, cells were grown and purified as described but buffers were adjusted to pH 7.0. Eluted protein was treated with a Roti-Spin Mini-10 MWCO(KD) to adjust conditions to 50 mM HEPES/KOH, pH 7.5. Protein concentration was set to 10 µg µl^–1^. To allow for autophosphorylation, 100 µg of purified H_6_-PSKR1-KD were incubated for 1 h at 25 °C in 50 mM HEPES-KOH, pH7.5, 10 mM MnCl_2_, 1 mM DTT, and 0.2 mM ATP in a total volume of 250 µl. Prior to the pull-down with MaBP-FLAG-CaM2, autophosphorylated PSKR1-KD was dialyzed to 20 mM Tris, 200 mM NaCl at pH7.5.

Interaction between H_6_-PSKR1-KD and MaBP-FLAG-CaM2 was analyzed by incubating samples on a rotator for 30 min at RT, followed by another incubation step as mentioned above with washed 25 µl amylose resin (NEB) in a Pierce® Spin Column (Thermo Scientific). The amylose resin was centrifuged for 1 min at 400 *g* at RT, washed three times with 300 µl 20 mM Tris-HCl, pH 7.5, 200 mM NaCl with 100 µM CaCl_2_ or 100 µM MgCl_2_ or without any divalent cations, as indicated. To elute MaBP-FLAG-CaM2 with interacting PSKR1-KD, the amylose resin was mixed with 50 µl of 20 mM Tris-HCl, pH 7.5, 200 mM NaCl, 10 mM maltose for 5 min and centrifuged for 1 min at 400 *g* at RT. The protein complex was separated by SDS-PAGE using a 12.5% gel. Proteins were blotted onto a PVDF membrane. MaBP-FLAG-CaM2 was detected by monoclonal anti-FLAG M2 antibodies (Sigma) and H_6_-PSKR1-KD or H_6_-PSKR1-KD(W831S) by anti-His (6x-His Epitope Tag Antibody His.H8, Thermo Fisher) antibodies. As secondary antibody, a horseradish peroxidase conjugated anti-mouse antibodies (Life Technologies) was used and visualized through ECL Plus Western Blotting substrate (Pierce).

### Homology modelling

A model of the cytoplasmic domain of PSKR1 was built with Phyre2 (Kelley *et al.*, 2015) by using the PDB templates 2QKW, 3TL8, 1OPL, 2FO0, 4XI2, and 4L68. A total of 93% of the sequence was modelled at >90% confidence and the first 22 amino acids of the juxtamembrane domain were modelled *ab initio*. The structure of the PSKR1 model was visualized by PyMOL version 1.2r 1 (Schrödinger, LLC). Definition of domains, such as the N-lobe, was done based on the model of BRI1 generated by Bojar *et al.* (2014). Secondary structures were named according to Taylor and Kornev (2011). Phosphorylations at specific residues in the model were added using the PyMol plugin PyTMs by Warnecke *et al.* (2014).

### Statistics

Statistical analysis of *in vitro* kinase activities was carried out with R (https://CRAN.R-project.org/doc/FAQ/R-FAQ.html). For all pairwise comparisons, a Kruskal–Wallis test with Bonferroni as *P*-value adjustment method (*α*=0,05) was run. Plant growth data were analyzed with a Kruskal–Wallis comparison against a control group with Dunn’s test as a *post hoc* test using a macro in Minitab (http://www.minitab.com).

## Results

### PSKR1 phosphosites regulate kinase activity in a site-specific manner

**Fig. 2.**
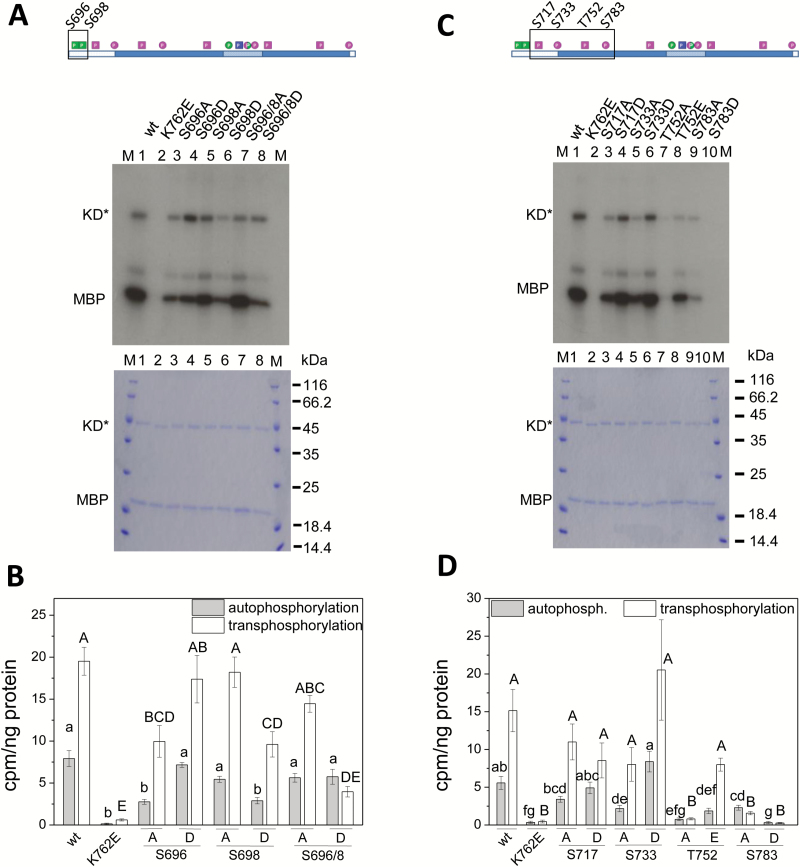
Phosphosites in the JM domain and in the N-lobe differentially alter PSKR1 kinase activity. The boxed residues in the juxtamembrane domain (A) and the N-terminal lobe (C) of PSKR1-KD (shown on the top) were point-mutated to either A as phosphoablative or D as phosphomimic mutations, and expressed as soluble PSKR1-KD (KD*) isoforms. A detailed description of the phosphosites is provided in Supplementary Fig. S2. Wild-type kinase (wt) and the inactive K762E variant were included as controls in each assay. (A, C) Kinase isoforms (0.25 µg) were incubated with ^32^P-ATP and 0.5 µg of the substrate MBP. The autoradiograph (top) shows auto- and transphosphorylation activities. A Coomassie-stained gel (bottom) shows loading of KD* and MBP, and M indicates the size marker in kDa. (B, D) Incorporated ^32^P was quantified by liquid scintillation. Auto- and transphosphorylation activities are shown as cpm ng^–1^ kinase isoform or ng^–1^ MBP. Results are means ±SE from three independent experiments with two replicates each. Significantly different values are indicated by different lower case letters for autophosphorylation and with capital letters for transphosphorylation (Kruskal–Wallis, *P*<0.05). (This figure is available in color at *JXB* online.)

**Fig. 3.**
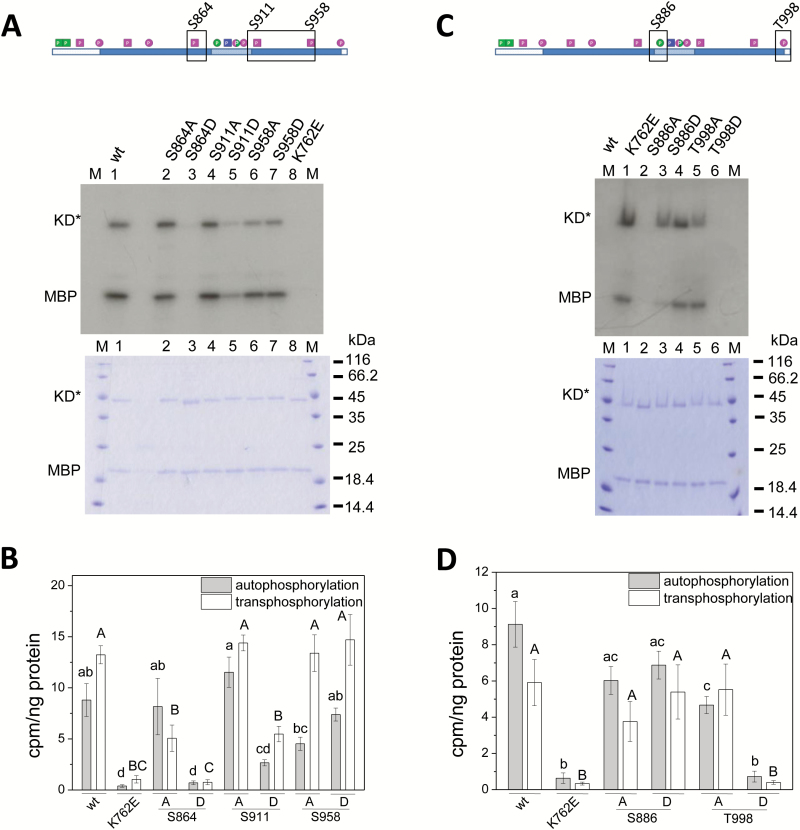
Differential impact of phosphosites in the C-lobe and at the CT on PSKR1 kinase activity. The boxed residues (A, C; top) in the N-lobe of PSKR1-KD were point-mutated to either A or D, and kinase activity was compared to the wild-type and to the inactive K762E isoform. (A, C) The kinase isoforms (0.25 µg) were incubated with ^32^P-ATP and 0.5 µg of the substrate MBP. The autoradiograph (top) shows auto- and transphosphorylation activities. A Coomassie-stained gel (bottom) shows loading of KD* and MBP, and M indicates the size marker in kDa. (B, D) ^32^P incorporated in PSKR1-KD and MBP was quantified and analyzed as described in the legend for Fig.2B and 2D. Significantly different values are indicated by different lower case letters for autophosphorylation and with capital letters for transphosphorylation (Kruskal–Wallis, *P*<0.05; B, *n*=6; D, *n*=8,). (This figure is available in color at *JXB* online.)

To evaluate the impact of a defined phosphorylation on PSKR1 kinase activity, we expressed soluble kinase variants with phosphosites replaced by an unphosphorylatable alanine on the one hand or by an aspartate or glutamate on the other hand to mimic a phosphorylated serine or threonine. The kinase variants were ectopically expressed in *E. coli*, affinity-purified via their N-terminal His-tag, and analyzed for *in vitro* kinase activity (Figs 2 and 3). Myelin basic protein (MBP) was added as a kinase substrate, allowing us to monitor autophosphorylation of the kinase and transphosphorylation of MBP at the same time. As controls, we included the wild-type kinase and the K762E isoform in each assay. The Lys (K762 in PSKR1) in the AxK motif of the β3-strand of the N-lobe forms a salt bridge with a Glu in the αC-helix (E778 in PSKR1) that engages in binding of the α- and β-phosphates of ATP (Huse and Kuriyan, 2002) (Fig. 1A, B). Mutating the conserved K to E abolishes kinase activity (Taylor and Kornev, 2011; Fig. 2). Kinase activities were visualized by autoradiography (Figs 2 and 3). In addition, autophosphorylated kinase and transphosphorylated MBP were quantified by liquid scintillation counting of incorporated ^32^P (Figs 2 and 3).

S696 and S698 in the JM domain are phosphorylated *in planta* (Hartmann *et al.*, 2015). Regulation of kinase activity by phosphorylation at these sites was studied by generating six PSKR1 kinase variants that were mutated at either one of the two sites or at both phosphorylation sites (Fig. 2A). Mutating S696 to alanine reduced kinase activity whereas the S696D variant had wild-type activity (Fig. 2A, B). The S698 site showed an inverse impact. Since both S696 and S698 are phosphorylated *in planta*, we analyzed kinase isoforms with both sites mutated. Interestingly, the unphosphorylatable S696A/S698A variant had wild-type activity while the S696D/S698D variant had wild-type autophosphorylation activity but was impaired in transphosphorylation activity (Fig. 2A, B), suggesting that the JM residues S696 and S698 are involved in substrate recognition and binding or phosphotransfer to the substrate. Taken together, our data indicate that phosphorylation within the JM domain regulates PSKR1 transphosphorylation activity. By contrast, autophosphorylation activity is independent of the JM phosphorylation status.

Four phosphorylated residues were identified in the N-lobe of the PSKR1 kinase (that extends up to Y807) (Fig. 1). S717 and S733 are located within the flexible loops of the N-lobe (Fig. 1A). Mutating S717 to either A or D did not significantly alter auto- or transphosphorylation activity (Fig. 2C, D). S717 is present in PSKR1 from *Arabidopsis thaliana* but not in PSKR1 orthologs from other plants (Hartmann *et al.*, 2015) (Supplementary Fig. S2). It is hence conceivable that this site has not acquired a detectable role in kinase regulation. S733 and T752 frame the Gly-rich loop with the GxGxxG-motif that participates in positioning of the adenine moiety and the γP of ATP for catalysis (Kornev and Taylor, 2015). Phosphorylation of S733 favors kinase activity as the S733D variant was more active than the S733A kinase (Fig. 2C, D). T752 is located in the β2 strand of the N-lobe (Fig. 1A), a highly conserved secondary structure of kinases. The T752A mutation abolished kinase activity, indicating that phosphorylation of this residue is crucial for receptor activation (Fig. 2C, D). Interestingly, the T752E variant was unable to autophosphorylate but did retain transphosphorylation activity.

A fourth phosphosite was identified in the ATP-binding region of the N-lobe at S783. This serine in αC is highly conserved in higher plant PSKR1 orthologs (Hartmann *et al.*, 2015) (Supplementary Fig. S2). The charged residues corresponding to K762 and E778 in PSKR1 form a salt bridge that is a hallmark of an active kinase. A negative charge at S783 might interfere with the ionic interaction between K762 and E778 (Fig. 1B). However, the S783A isoform also showed strongly reduced autophosphorylation and no significant transphosphorylation activity, suggesting that S783 is a phosphorylatable residue with invariable structural characteristics.

In the C-lobe, seven phosphorylation sites were identified, of which four sites within the activation segment were characterized previously (Hartmann *et al.*, 2015). Of the three as yet uncharacterized sites, S864 is located within the catalytic loop in subdomain VIb that is N-terminal to the activation segment (Fig. 1A, C). S864 is invariant in all PSKR1 and PSKR2 orthologs (Hartmann *et al.*, 2015) (Supplementary Fig. S2), suggestive of a highly conserved function. The phosphomimic S864D replacement abolished kinase activity, suggesting that phosphorylation of S864 is an efficient way of inactivating the receptor (Fig. 3A, B). L867 in β7 is a functional residue of the catalytic spine and interacts with the adenine ring of ATP (Taylor and Kornev, 2011). It is conceivable that a negative charge at S864 interferes with this hydrophobic interaction (Fig. 1C). The S864A mutation did not alter autophosphorylation activity but partially impaired transphosphorylation activity, supporting a role of S864 in substrate phosphorylation.

S911 is located at the protein surface in close proximity to S893 and S894 in the activation segment (Fig. 1D). Point mutations of S911 revealed a reduced kinase activity of S911D over S911A or wild-type kinase with regard to both auto- and transphosphorylation activity, supporting the conclusion that S911 phosphorylation is a mechanism to regulate PSKR1 activity (Fig. 3A, B). By contrast, neither the S958A nor the S958D mutation affected kinase activity significantly. S911 is highly conserved among PSKR1 orthologs whereas S958 is present only in *Arabidopsis thaliana* (Hartmann *et al.*, 2015) (Supplementary Fig. S2), arguing for a recently acquired non-functional phosphosite.

The CT of PSKR1 was found to be phosphorylated at T998 *in vitro*. This phosphosite is located in the αI helix. The T998 side-chain points to the highly conserved αE helix that harbors the CaM binding site (Fig. 1A, E). Two aliphatic leucine residues (L845 and L846) in the αE helix flank the polar T998 side-chain (Fig. 1E). Introduction of a negative charge at this site by a T998D substitution rendered the kinase completely inactive while the unphosphorylatable T998A isoform had wild-type activity. This result was confirmed in the T998E isoform where the threonine at position 998 was replaced by a glutamic acid that is more similar in size to threonine (Supplementary Fig. S3). These results demonstrated that phosphorylation of the C-terminus acts as an on/off switch for PSKR1 kinase activity.

### *Phosphosite mutagenesis of PSKR1 reveals organ-specific receptor regulation* in planta

**Fig. 4.**
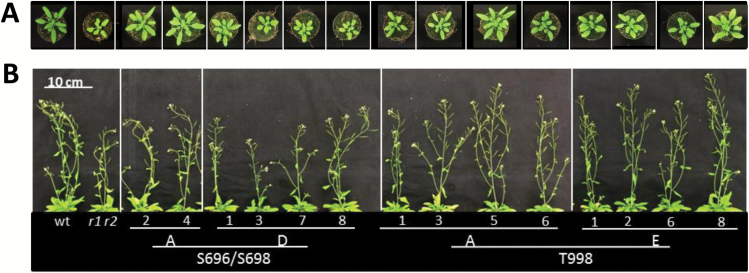
Phosphorylation in the JM domain and at the CT impair shoot growth. The full-length PSKR1 receptor was mutated as indicated and introduced into the *pskr1-3 pskr2-1* receptor null background. Plants were grown on soil for (A) 4 weeks to measure rosette areas, and (B) for 6 weeks to measure plant height. The numbers indicate independently transformed lines. (This figure is available in color at *JXB* online.)

**Fig. 5.**
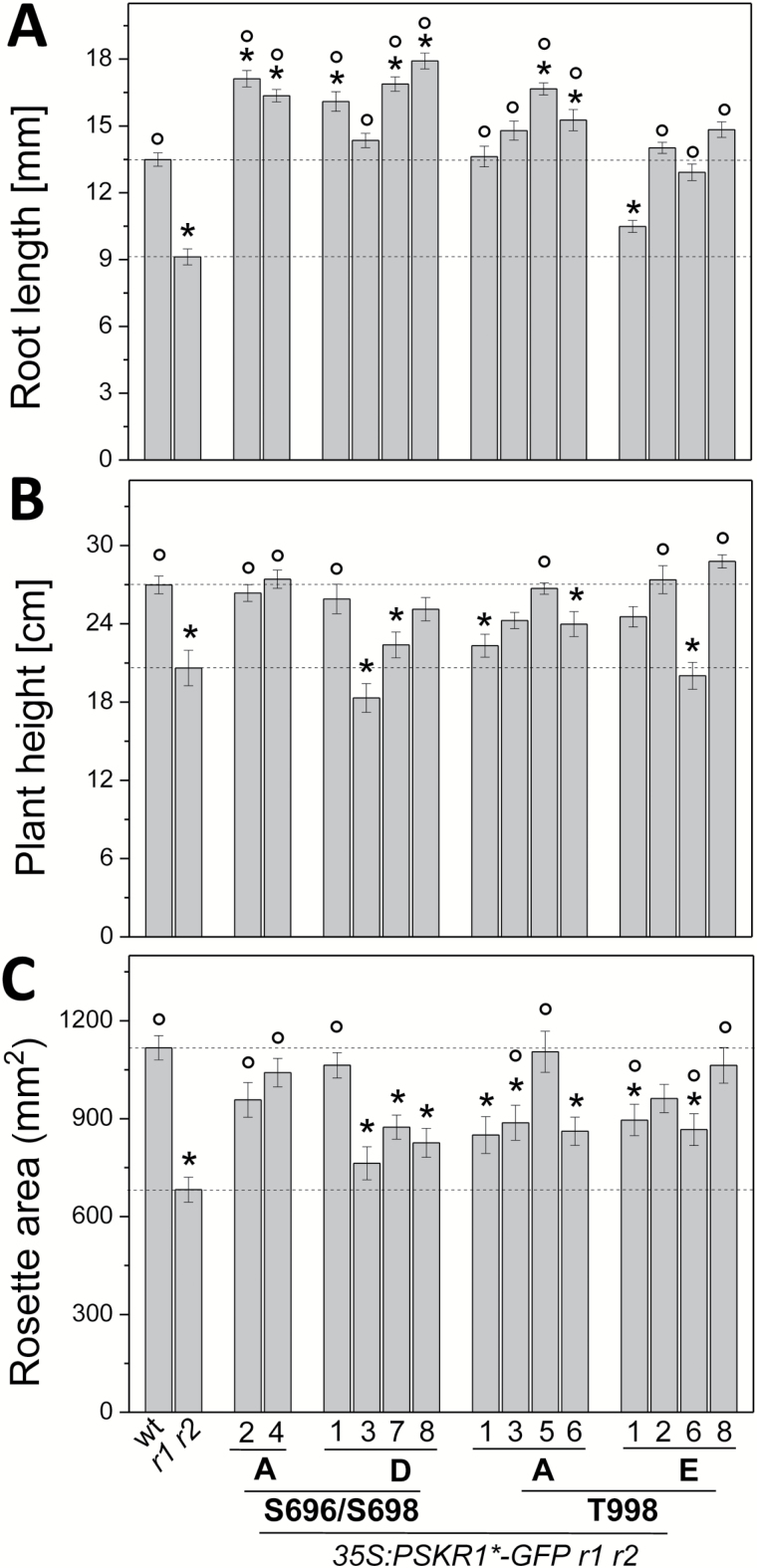
Phosphorylations at the JM domain and CT differentially affect PSKR1 receptor activity in roots and shoots. Full-length PSKR1 receptor isoforms as indicated were expressed in the *pskr1-3 pskr2-1* receptor null background, with different numbers indicating independent lines. (A) Length of main root of 5-d-old seedlings grown under sterile conditions (*n*=35–94). (B) Plant height of 6-week-old soil-grown plants (*n*=18–27). (C) Projected rosette area of 4-week-old soil-grown plants (*n*=19–27). Values are means from three independent biological experiments. Significant differences compared to the wild-type (wt) are indicated by * and significant differences compared to *pskr1-3 pskr2-1* (*r1 r2*) are indicated by ° (Kruskal–Wallis with Dunn’s test as *post-hoc*; *P*<0.05).

To study the biological function of phosphosites within the JM and CT domains we expressed mutated full-length receptor variants in the PSK receptor null background. We analyzed rosette area and plant height (Fig. 4) as well as primary root lengths of several independent lines per genotype (Figs 4 and 5). Surprisingly, the phenotypes that we observed were not consistent between root and shoot, and the kinase activities did not correlate with plant phenotypes in each case. Expression of *35S:PSKR1-GFP* in the null background was shown previously to rescue growth of both root and shoot. In both studies, transcript levels were lower than in the wild-type (Hartmann *et al.*, 2014) (Supplementary Fig. S5).

Specifically, roots expressing the PSKR1(S696A/S698A) receptor under the control of the 35S Cauliflower Mosaic Virus promoter showed an overexpression phenotype with longer roots than in the wild-type (Fig. 5A) whereas plant height and rosette areas were comparable to the wild-type (Figs 4A, B and 5B, C). These results are in agreement with wild-type kinase activity *in vitro* of the respective kinase isoform and suggest that receptor activity is limiting in roots but not in shoots. The PSKR1(S696D/S698D) receptor isoform resulted in an overexpression phenotype in the roots (Fig. 5A) but not in the shoot where growth was reduced compared to the wild-type (Fig. 5B, C). The phosphomimic S696D/S698D kinase isoform has reduced transphosphorylation activity. Our data hence suggest that transphosphorylation activity is limiting in shoots but not in roots, pointing to organ-specific or development-dependent receptor regulation *in planta*.

The T998D as well as the T998E variants were kinase-inactive *in vitro*, indicating that phosphorylation at T998 within the CT inhibits PSKR1 kinase activity (Fig. 3C, D, Supplementary Fig. S3). *In planta*, roots of PSKR1(T998E) seedlings had wild-type length in three of four independent lines but were on average shorter than PSKR1(T998A) roots (Fig. 5A). Hence, unlike T998E kinase activity *in vitro*, PSKR1(T998E) receptor activity *in planta* was not abolished, pointing to receptor regulation beyond phosphorylation *in situ*. In contrast to roots, shoot growth of PSKR1(T998A) plants was reduced in three of four lines. PSKR1(T998E) plants had an intermediary shoot phenotype compared to the wild-type and compared to PSK receptor null plants, again indicating that this phosphorylatable threonine residue plays a different role in roots and shoots. While in roots the phosphorylation status alters receptor activity, this was not observed in the shoot. This suggests that T998 rather than its phosphorylation status is required.

In summary, *in vitro* kinase activities and *in planta* receptor activities do not correlate for each isoform analyzed, indicating additional levels of receptor regulation. Furthermore, the activity of particular receptor isoforms differs in the roots and shoot, suggesting that organ-specific or development-dependent factors influence receptor signaling. PSKR1 can hence be modified at levels other than, and possibly independent of, specific phosphorylation events.

### Regulation of PSKR1 kinase by calmodulin

**Fig. 6.**
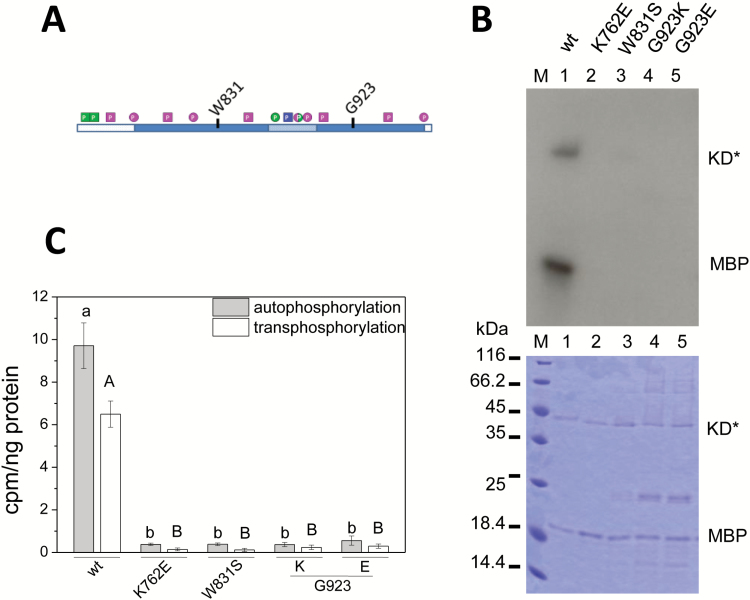
Mutation of the calmodulin binding site abolishes PSKR1 kinase activity. A conserved tryptophan (W831) in the calmodulin binding site of PSKR1-KD was mutated to a serine. Included as controls were the wild-type (wt), the inactive K762E, and two isoforms in which G923 within the predicted guanylyl cyclase center was mutated to lysine or glutamate. (A) Schematic drawing of PSKR1-KD highlighting the point mutations analyzed. (B) The kinase isoforms (0.25 µg) were incubated with ^32^P-ATP and and 0.5 µg of the substrate MBP. The autoradiograph (top) visualizes auto- and transphosphorylation activities of the isoforms (KD*). The Coomassie-stained gel (bottom) shows protein loading. M indicates the size marker in kDa. (C) Incorporated ^32^P was quantified by liquid scintillation. Auto- and transphosphorylation activities are shown as cpm ng^–1^ kinase isoform or ng^–^ MBP. Results are means ±SE from three independent experiments with two replicates each. Significantly different values (Kruskal–Wallis, *P*<0.05) are indicated by different lower case letters for autophosphorylation and capital letters for transphosphorylation. (This figure is available in color at *JXB* online.)

The kinase domains of many receptor kinases including PSKR1 are folded in their active state *in vitro*, which is in fact the basis for any *in vitro* kinase assay. If a kinase can auto-activate itself not only *in vitro* but also *in planta* then mechanisms are needed to keep the kinase in check. The calcium sensor calmodulin (CaM), which has been shown previously to interact with PSKR1 (Hartmann *et al.*, 2014), might have such a regulatory function. Mutation of a conserved hydrophobic tryptophane to a hydrophilic serine (W831S) in the predicted CaM binding αE helix (Figs 1A and 6A) abolishes CaM binding and PSKR1 receptor activity (Hartmann *et al.*, 2014). To understand the role of CaM binding for PSKR1 kinase activity we analyzed the soluble PSKR1(W831S) kinase isoform. As controls we included wild-type kinase, the inactive K762E isoform, and two kinase isoforms that were mutated at G923 within the predicted guanylyl cyclase center in ɑF of PSKR1 (Fig. 1A) (Kwezi *et al.*, 2011) to either a lysine (G923K) or a glutamate (G923E). The W831S point mutation abolished both auto- and transphosphorylation activity *in vitro*. This is unexpected as kinase activity was measured in the absence of CaM. It suggests that W831 is essential for kinase activity independent of CaM binding, possibly for structural reasons (Fig. 6B, C). A similar loss of activity was observed for the G923K and G923E isoforms, supporting the view that G923 is an essential amino acid irrespective of its proposed role in cGMP formation.

**Fig. 7.**
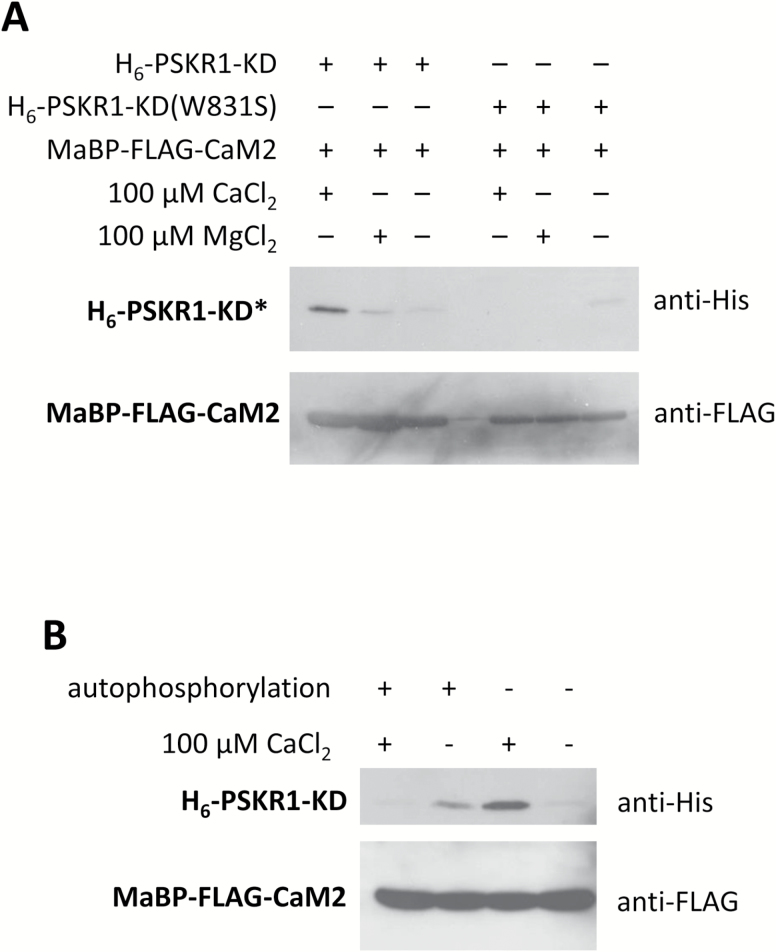
Binding of CaM2 to PSKR1-KD is Ca^2+^-dependent and determined by the phosphorylation state of PSKR1-KD. (A) Western blots with His-tagged (H_6_) PSKR1-KD (H_6_-PSKR1-KD) or H_6_-PSKR1-KD(W831S) that was bound by maltose binding protein (MaBP)-tagged and FLAG-tagged CaM2 (MaBP-FLAG-CaM2) in the presence of 100 µM CaCl_2_ or 100 µM MgCl_2_ or without divalent cation. (B) H_6_-PSKR1-KD was incubated with ATP to allow for autophosphorylation or left unphosphorylated prior to pull-down with MBP-FLAG-CaM2 in the presence or absence of 100 µM Ca^2+^. Blots are shown in greyscale and were uniformly adjusted in contrast (–20%) and brightness (+40%). Results were confirmed in independent experiments.

We next studied the impact of calcium on binding of CaM2 to PSKR1-KD using pull-down assays. CaM2/PSKR1-KD binding was stronger in the presence of Ca^2+^ than with Mg^2+^, supporting specificity of this interaction (Fig. 7A). No interaction occurred between CaM2 and PSKR1-KD(W831S), as demonstrated previously by Bimolecular Fluorescence Complementation (Hartmann *et al.*, 2014). We next studied whether binding of CaM was influenced by phosphorylation of PSKR1-KD. Ectopically expressed PSKR1-KD is likely to be partially phosphorylated in *E. coli*, as suggested by a shift in mobility after dephosphorylation (Supplementary Fig. S4). Incubation of hypophosphorylated PSKR1-KD with ATP resulted in autophosphorylation whereas without ATP PSKR1-KD remained in its hypophosphorylated state. Subsequently, binding to CaM2 was analyzed in the absence or presence of calcium. The strongest interaction was observed between Ca^2+^-CaM2 and hypophosphorylated PSKR1-KD (Fig. 7B) while autophosphorylation prevented binding of Ca^2+^-CaM. In summary, the interaction of CaM2 and PSKR1 is Ca^2+^-dependent and Ca^2+^-CaM binds preferentially to hypophosphorylated PSKR1.

### PSKR1 kinase activity is not regulated by Ca^2+^-CaM2

**Fig. 8.**
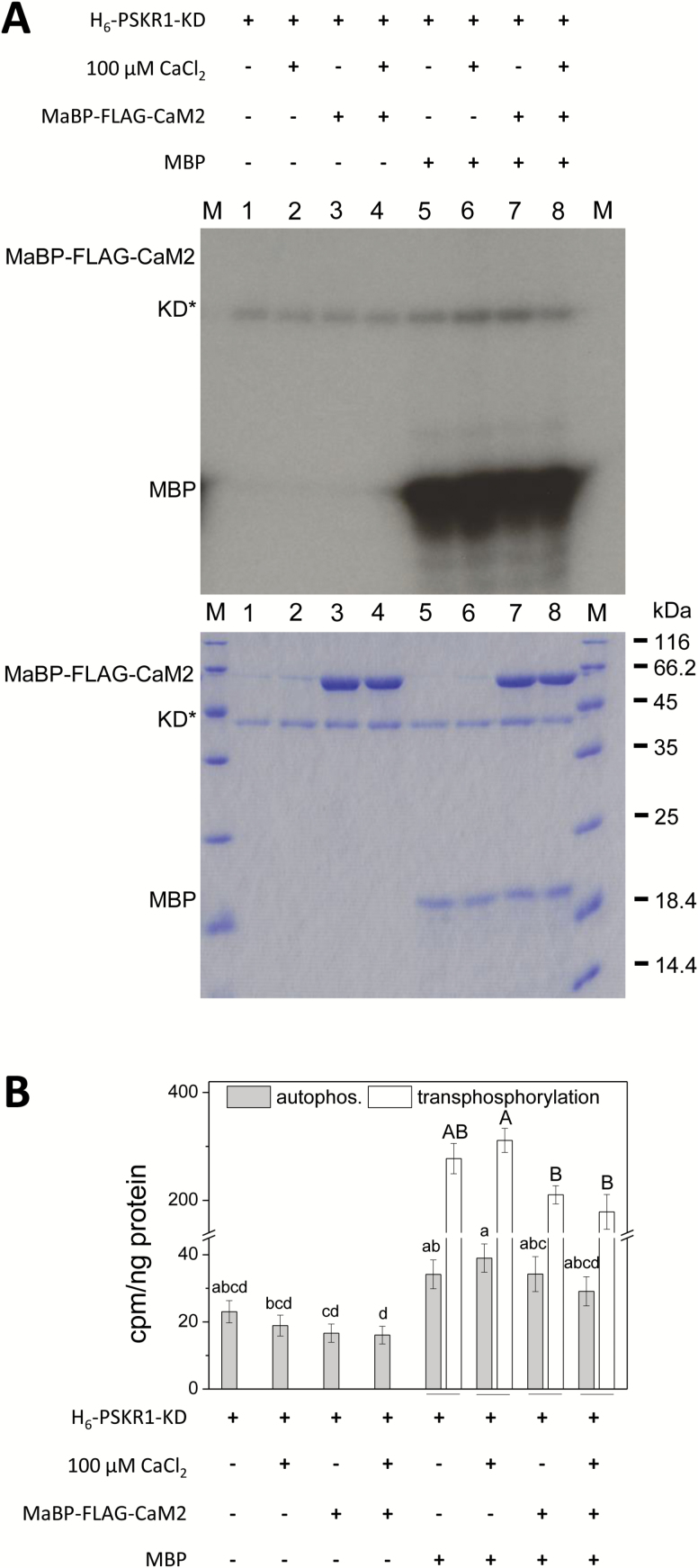
PSKR1 kinase activity is not altered by binding of Ca^2+^-CaM2. (A) PSKR1-KD (0.25 µg) was incubated with MaBP-FLAG-CaM2 (1.5 µg) in the presence of 100 µM Ca^2+^ or without calcium to allow for Ca^2+^-CaM/PSKR1-KD binding, followed by a kinase assay with ^32^P-ATP and 0.5 µg of the substrate MBP. The autoradiograph (top) visualizes auto- and transphosphorylation activities. The Coomassie-stained gel (bottom) shows PSKR1-KD, MaBP-FLAG-CaM2, and MBP; M indicates the size marker in kDa. (B) Incorporated ^32^P was quantified by liquid scintillation. Auto- and transphosphorylation activities are shown as cpm ng^–^ kinase isoform or ng^–1^ MBP. Results are means ±SE of three independent biological experiments with two technical replicates each. Significantly different values are indicated by different lower case letters for autophosphorylation and with capital letters for transphosphorylation (Kruskal–Wallis, *P*<0.05). (This figure is available in color at *JXB* online.)

To study a possible role of calmodulin in regulating PSKR1 kinase activity, we compared kinase activity of PSKR1 when pre-incubated with Ca^2+^, CaM2, or Ca^2+^-CaM (Fig. 8). Pre-incubation was performed under the same conditions that were used for pull-down assays (Fig. 7). Kinase activity was measured in the presence or absence of the substrate myelin basic protein (MBP) as documented in autoradiographs (Fig. 8A) and quantified by liquid scintillation counting (Fig. 8B). Unexpectedly, autophosphorylation activity was somewhat higher in the presence of MBP, possibly indicating structural rearrangements following occupation of the substrate binding site that favor autophosphorylation. Ca^2+^-CaM2 did not significantly alter kinase activity. Our analysis further revealed that CaM2 itself is not phosphorylated by PSKR1 (Fig. 8A).

Taken together, CaM2 binds to hypophosphorylated PSKR1 in a calcium-dependent manner. Ca^2+^-CaM2, however, does not regulate kinase activity of PSKR1-KD *in vitro*.

## Discussion

The peptide receptor PSKR1 is a highly phosphorylatable receptor kinase with phosphosites present within the kinase proper as well as in the JM and CT domains. Fourteen phosphosites were identified by our group (Hartmann *et al.*, 2015), of which six, S696, S698, S864, S886, S893, and T998, were confirmed in an independent study in which three more sites were found (Fig. 1A and Supplementary Fig. S2; Mitra *et al.*, 2015). S886, T890, S893, and S894 are located in the activation segment that includes the AL as a hallmark of RD kinases. The activation segment forms a loop across the substrate binding cleft, thereby preventing access for the substrate. Phosphorylation of activation segment residues causes a conformational change that allows substrate binding (Johnson *et al.*, 1996). Site-directed mutagenesis of single and multiple phosphosites in the activation segment of PSKR1 confirmed that this regulatory mechanism also works in PSKR1 (Hartmann *et al.*, 2015). Our observation that these phosphosites are evolutionarily highly conserved in PSKR1 orthologs supports a conserved function (summarized in Supplementary Fig. S2).

The multiple phosphosites outside of the activation segment are indicative of additional regulatory mechanisms that may target not only kinase activity. Receptor protein kinases are optimized to transmit a signal rather than for catalytic activity. Their task is to activate downstream effectors, initiate and terminate a signal or integrate multiple signals. Differential phosphorylation of receptor kinases may hence determine substrate specificity and interactions with other proteins that serve cross-talk with other signaling pathways, as well as degradation or internalization of the receptor to terminate a signal. Analysis of *in vitro* and *in vivo* activity of PSKR1 phosphosite isoforms is a useful first step to unravel such functions.

### Phosphosites in ATP binding domains are crucial for kinase activity

In PSKR1, S733 and T752 flank the glycine (G)-rich loop near the ATP binding region. The phosphomimic substitution S733D resulted in enhanced kinase activity over the S733A variant. The T752E variant was likewise more active than the unphosphorylatable T752A isoform, whereas autophosphorylation ability was impaired even in the phosphomimic isoform. In the related LRR receptor kinase BRI1 an S891D mutation within the G-rich loop resulted in severe dwarfism (Oh *et al.*, 2012), indicative of receptor inactivation. The S891A mutation of BRI1 caused hyperactivity of BRI1, resulting in seedling growth promotion. The authors concluded that S891 phosphorylation is a reversible means to deactivate BRI1. The finding that unphosphorylatable S733 and T752 reduced kinase activity suggests that the G-rich loop may be a target for receptor kinase regulation in general, with different modes acting in different receptor kinases.

A conserved Glu (E778 in PSKR1) in αC interacts with a conserved Lys (K762 in PSKR1) in β3 at the ATP binding site (Fig. 1B) (Huse and Kuriyan, 2002). When the activation segment is in its dephosphorylated state the E–K ion pair is disrupted, resulting in impaired ATP binding and lowered rates of phosphotransfer to the substrate (Huse and Kuriyan, 2002; Adams 2003). The phosphosite S783 is in αC, which is known as a dynamic regulatory element. Its position is crucial for efficient catalysis (Taylor and Kornev, 2011). S783 is a nearly invariant amino acid in PSKR1 orthologs that is close to the E–K pair (Supplementary Fig. S2) (Hartmann *et al.*, 2015). A charge at this Ser may disrupt the salt bridge, as suggested by the inactive kinase isoform S783D. However, the S783A also had strongly reduced kinase activity, arguing for an invariant residue the phosphorylation of which may act as an on/off switch.

S864 within the catalytic loop in subdomain VIb C-terminal to the RD motif (R859/D860) is an invariant amino acid in PSKR1 orthologs (Supplementary Fig. S2). The S864A isoform had wild-type autophosphorylation activity and reduced transphosphorylation activity. The S864D isoform was kinase-inactive, possibly indicating a role for S864 phosphorylation in regulating the catalytic cycle. Mutation of S911 suggested attenuation of kinase activity by phosphorylation. The side-chain of S911 points in the direction of the activation segment phosphosites S893 and T894, and this possibly causes a change of activation segment orientation. A similar situation was shown for BRI1 with T1039, S1042, and S1060 (Bojar *et al.*, 2014). In contrast, mutation of S958 did not significantly affect kinase activity. The S958 phosphorylation site is unique to *Arabidopsis thaliana* (Hartmann *et al.*, 2015) and is located in a flexible loop on the protein surface. It is conceivable that this phosphosite has evolved only recently and has not acquired a function with respect to kinase activity.

### PSKR1 regulation by phosphorylation at the extremes

The large number of phosphosites that were identified in the cytosolic PSKR1 receptor part point to an elaborate mode of PSKR1 regulation by reversible receptor modification. Phosphosites were found not only within the kinase proper but also in the JM (S696, S698) and CT (T998) domains, which are *per se* not required for kinase function. The 3D homology model of PSKR1-KD revealed close proximity of the JM residues S696 and S698 to the ATP binding cleft (Fig. 1A, Supplementary Fig. S1). Phosphomimic modification of both residues inhibited transphosphorylation activity *in vitro*. S696 and S698 are phosphorylated *in planta* and hence are biologically functional sites. At least one of the two sites has a Ser or Thr conserved in 83% of higher plant PSKR1 orthologs, supporting the idea that these sites confer a crucial function in PSKR1 signaling. LRR RLKs function in many diverse physiological processes. Phosphorylation of JM and CT regions is one way to overcome this functional diversity and to achieve specificity. Deletion of the BRI1 JM domain abolished the signaling function of the receptor whereas phosphorylation of the JM domain activated BRI1 (Wang *et al.*, 2005, 2008). Replacing S696/S698 in PSKR1 with the negatively charged amino acid Asp reduced transphosphorylation but not autophosphorylation activity *in vitro*, pointing to regulation of substrate binding via the JM. *In planta*, the growth-impaired phenotype of PSK receptor null plants was not, or only partially, rescued by the PSKR1(S696D/S698D) variant with regard to the shoot, indicating that shoot growth may be limited by transphosphorylation activity of PSKR1. By contrast, primary root growth was promoted by both the PSKR1(S696A/S698A) and the PSKR1(S696D/S698D) isoforms, indicating that growth-promoting activity of PSKR1 in the root is independent of JM phosphorylation. These findings are in agreement with the idea that phosphorylation at the JM domain regulates defined signal outputs that can be assigned to different organs such as roots and shoot, or to different developmental stages.

The *PSKR1* gene is differentially regulated in roots and shoot, as indicated by gene expression data summarized in the eFP browser (Winter *et al.*, 2007). Expression is induced by cold and salt stress (150 mM NaCl) in the roots but not the shoot, whereas expression increases in the shoot but not in the roots in response to osmotic stress (300 mM mannitol) (Kilian *et al.*, 2007), supporting the idea that PSKR1 activity is differentially regulated in roots and shoots in response to environmental signals. Differential phosphorylation of PSKR1 in roots and shoots may be yet another level of regulating the intensity or quality of the signal output. Different effects of distinct phosphosite mutations in roots and shoots seem plausible in light of the functional diversity of PSKR1 signaling.

While the JM seems to control the signal output by influencing substrate recognition, binding, or phosphotransfer, the CT acts as an on/off switch for kinase activity *in vitro*, with loss of activity in the T998D and T998E isoforms. A similar auto-inhibitory mechanism was observed in other receptor kinases. Deletion of the BRI1 CT domain was reported to increase kinase activity of BRI1 *in vitro* and *in planta* (Wang *et al.*, 2005), suggesting a conserved role in kinase regulation. Interestingly, plants expressing the PSKR1(T998E) isoform had reduced shoot growth but wild-type root lengths. These phenotypes are compatible with a PSKR1 receptor that is inactive in the shoot due to phosphorylations of S696/S698 at the JM domain and cannot be further inactivated via CT phosphorylation while the phosphorylation status of PSKR1 in the root may be different, allowing for inhibition by CT phosphorylation *in planta*.

### Binding of CaM to PSKR1 is calcium-dependent and occurs preferentially to hypophosphorylated PSKR1

Aside from acting as kinases, receptor kinases can have non-catalytic functions such as scaffolding activity (Kung and Jura, 2016). PSKR1 was shown to interact with the co-receptor BAK1, with the proton pumps AHA1 and AHA2 (Arabidopsis H^+^-ATPase1 and 2) (Ladwig *et al.*, 2015), and with calmodulins (CaM) (Hartmann *et al.*, 2014). BAK1 and AHAs in turn interact with the cation channel CNGC17 (cyclic nucleotide-gated channel 17), establishing a physical link between cell wall acidification and cation uptake (Ladwig *et al.*, 2015). CNGC17 is likely to permeate mono- and divalent cations including Ca^2+^. It is hence possible that PSKR1 activity brings about a rise in intracellular calcium levels that will result in the activation of CaM and binding of Ca^2+^-CaM to hypophosphorylated PSKR1.

Similar to PSKR1, BRI1 has a CaM binding site in subdomain VIa and a similar interaction of calmodulins with BRI1 was previously reported by Oh *et al.* (2012), suggesting that CaMs may have a conserved function in LRR-RLK signaling. However, co-expression of BRI1 with CaM in *E. coli* suppressed phosphorylation of *E. coli* proteins (Oh *et al.*, 2012) whereas the *in vitro* studies described here did not reveal a significant inhibition of kinase activity by Ca^2+^-CaM. These differing results may be due to the different experimental set-ups or may reflect actual differences in the regulation of PSKR1 and BRI1. The fact that Ca^2+^-CaM preferentially binds to the hypophosphorylated BRI1 and PSKR1 receptor kinases suggests that CaM has an as yet unexplored function in PSKR1 signaling, localization, stability, recycling, or complex assembly. It will be of interest to compare the phosphorylation patterns of the hypophosphorylated Ca^2+^-CaM2 binding with that of the *in vitro* autophosphorylated PSKR1-KD. Functional analyses of these sites will help identify the crucial phosphosite(s) that engage(s) in regulating Ca^2+^-CaM2/PSKR1 interaction.

Taken together, our study showed that differential phosphorylation of the peptide receptor PSKR1 is an efficient means to control receptor activity. The multitude of phosphosites identified suggests that phosphorylation may serve in integration of other signal pathways and/or specification of signal output. Root and shoot growth is affected in a differential manner by defined phosphosite mutations, supporting this view. PSK signaling also modifies pathogen responses (Mosher *et al.*, 2013) and differentiation (Rodiuc *et al.*, 2016). It is hence conceivable that different physiological outputs are mediated by differential receptor phosphorylation. In addition, PSKR1 is subject to control by the calcium sensor CaM. CaM2 binds to hypophosphorylated PSKR1, which, however, does not alter PSKR1 kinase activity. This interaction may influence, for example, receptor turnover or complex assembly, but its biological function has yet to be clarified.

## Supplementary Material

Supplementary Data
