# Supplementary material for: Phosphorylation of the phytosulfokine peptide receptor PSKR1 controls receptor activity
Source: J Exp Bot. 2017 Feb 23;68(7):1411–23. doi: 10.1093/jxb/erx030 (PMC5441923; doi:10.1093/jxb/erx030)
Supplement: Supplementary Data [file erx030_Supplementary_Data.zip › supplementary_table_S1.pdf]

Supplemental Table

Table 1 Oligonucelotides used to introduce point mutations in PSKR1.

Point-mutation	domain	Oligonucleotide sequence (5' – 3')
S696A	KD	AAATTTGTCGACTCAGGAGAAGTTGATCCGGAGATAGAAGAAGCTGAGAGCATGAATCG
S696D	KD	AAATTTGTCGACTCAGGAGAAGTTGATCCGGAGATAGAAGAAGATGAGAGCATGAATCG
S698A	KD	AAATTTGTCGACTCAGGAGAAGTTGATCCGGAGATAGAAGAATCCGAGGCTATGAATCG
S698D	KD	AAATTTGTCGACTCAGGAGAAGTTGATCCGGAGATAGAAGAATCCGAGGATATGAATCG
S696/S698A	FL	GGAGATAGAAGAAGCTGAGGCTATGAATCGTAAAGAACTC
		GAGTTCTTTACGATTCATAGCCTCAGCTTCTTCTATCTCC
S696/S698D	FL	GGAGATAGAAGAAGATGAGGATATGAATCGTAAAGAACTC
		GAGTTCTTTACGATTCATATCCTCATCTTCTTCTATCTCC
S717A	KD	GGTTTTGTTTCAGGCTAATGATAAAGAG
		CTCTTTATCATTAGCCTGAAACAAAACC
S717D	KD	GGTTTTGTTTCAGGATAATGATAAAGAG
		CTCTTTATCATTATCCTGAAACAAAACC
S733A	KD	GGACTCAACAAATGCTTTTGATC
		GATCAAAAGCATTTGTTGAGTCC
S733D	KD	GGACTCAACAAATGATTTTGATC
		GATCAAAATCATTTGTTGAGTCC
T752A	KD	GGTTTACAAAGCAGCTTTACCAGACGG
		CCGTCTGGTAAAGCTGCTTTGTAAACC
T752E	KD	GGTTTACAAAGCAGAGTTACCAGACGG
		CCGTCTGGTAACTCTGCTTTGTAAACC
S783A	KD	GTTGAAACACTCGCTAGAGCACAGC
		GCTGTGCTCTAGCGAGTGTTTCAAC
S783D	KD	GTTGAAACACTCGATAGAGCACAGC
		GCTGTGCTCTATCGAGTGTTTCAAC
S864A	KD	CCGCGATATTAAATCGGCTAATATTCTTCTCGACG
		CGTCGAGAAGAATATTAGCCGATTTAATATCGCGG
S864D	KD	CCGCGATATTAAATCGGATAATATTCTTCTCGACG
		CGTCGAGAAGAATATTATCCGATTTAATATCGCGG
S886A	KD	CAAGGCTGATGGCTCCTTACGAGAC
		GTCTCGTAAGGAGCCATCAGCCTTG
S886D	KD	CAAGGCTGATGGATCCTTACGAGAC
		GTCTCGTAAGGATCCATCAGCCTTG
S911A	KD	CGGGCAAGCTGCTGTTGCTACTTAC
		GTAAGTAGCAACAGCAGCTTGCCCG
S911D	KD	CGGGCAAGCTGATGTTGCTACTTAC
		GTAAGTAGCAACATCAGCTTGCCCG
S958A	KD	TGAAGCATGAGGCTCGAGCAAGCGA
		TCGCTTGCTCGAGCCTCATGCTTCA
S958D	KD	TGAAGCATGAGGATCGAGCAAGCGA
		TCGCTTGCTCGATCCTCATGCTTCA
T998A	KD	AACGCTCCTAGGCTAGACATCATCAAGCCAAGAGACTAACTGTTGAGCCGTTGG
T998A	FL	GGTCGCGGCCGCCGCGACATCATCAAGCCAAGAGACTAACTGTTGAGCCGTTG
T998D	KD	AACGCTCCTAGGCTAGACATCATCAAGCCAAGAGACTAACTGTTGATCCGTTGG
T998E	FL	GGTCGCGGCCGCCGCGACATCATCAAGCCAAGAGACTAACTGTTGTTCCGTTGGCCTCT
