# Supplementary material for: Phosphorylation of the phytosulfokine peptide receptor PSKR1 controls receptor activity
Source: J Exp Bot. 2017 Feb 23;68(7):1411–23. doi: 10.1093/jxb/erx030 (PMC5441923; doi:10.1093/jxb/erx030)
Supplement: Supplementary Data [file erx030_Supplementary_Data.zip › Supplementary_figures_S2_S5.pdf]

Journal of Experimental Botany

Phosphorylation of the phytosulfokine peptide receptor PSKR1 controls kinase activity, binding to Ca^{2+}/CaM and receptor activity

Christine Kaufmann, Michael Motzkus, Margret Sauter

Supplementary Figure S2

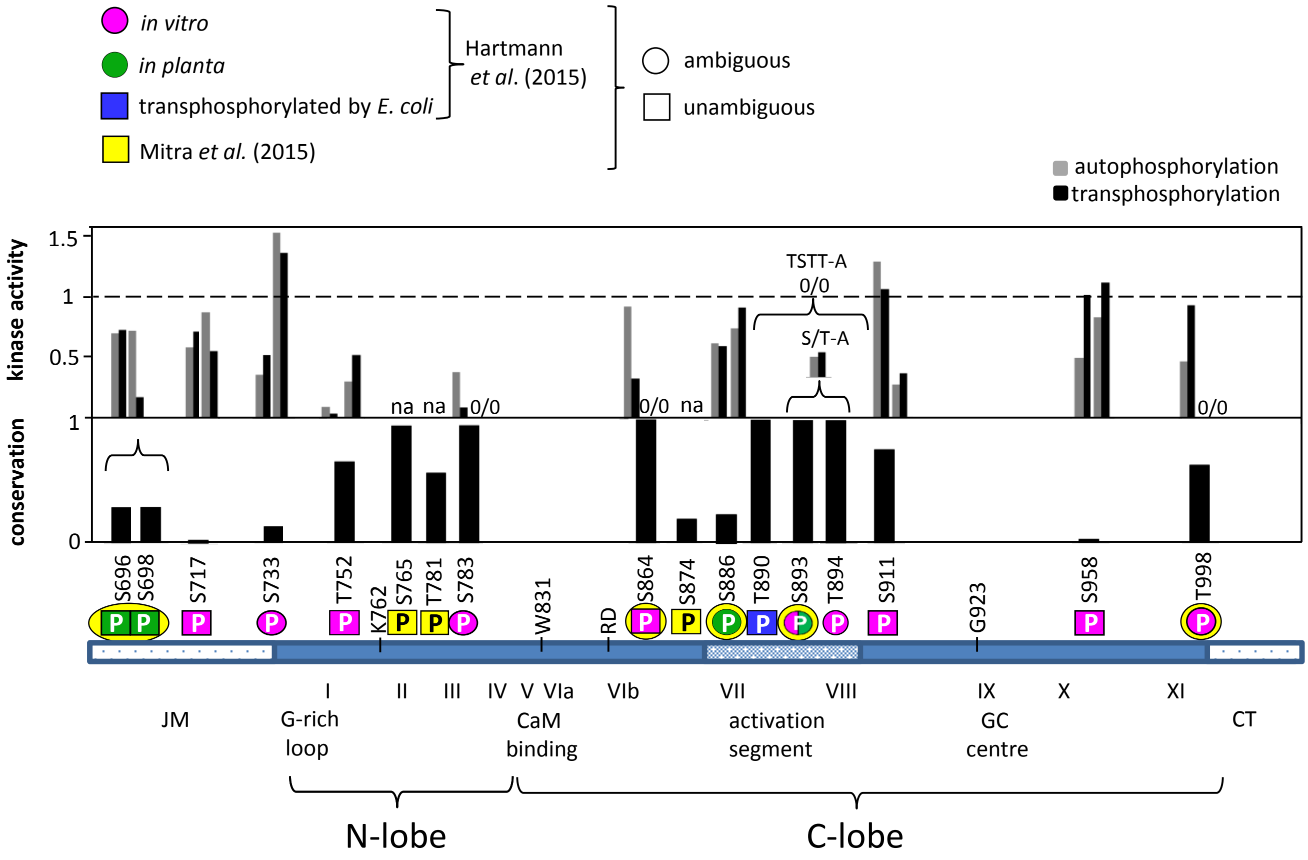

Supplementary Figure S2: Schematic overview of mapped PSKR1-KD phosphosites in *Arabidopsis thaliana* (Hartmann *et al.*, 2015; Mitra *et al.*, 2015) in relation to the 12 kinase subdomains and other functional sites referred to in the paper. The middle panel shows conservation of phosphosites in higher plant PSKR1 orthologs (taken from Hartmann *et al.*, 2015) with 1 indicating 100% conservation with S/T variations considered as conserved. The top panel shows the relative kinase activities of PSKR1-KD isoforms summarized from Figures 1, 2 and 5 and from Hartmann *et al.* (2015). At each phosphosite the two columns to the left show kinase activities of the phosphoablative A isoform; the two columns to the right show activities of the phosphomimetic D or E isoforms. 0: no activity; na: not analyzed. To allow for comparison among experiments, the kinase activities of the K762E isoform were set to zero and those of wild type were set to one in each case.

Supplementary Figure S3

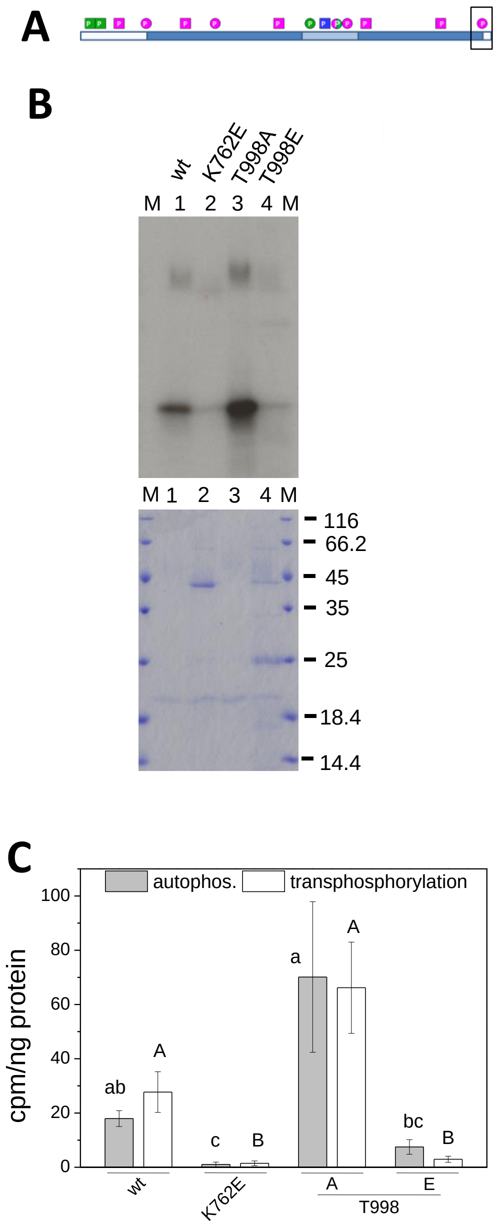

Supplementary Figure S3: Phosphomimetic T998E mutation abolishes kinase activity. Auto- and transphosphorylation activities of the wild type PSKR1-KD (wt), the inactive PSKR1-KD(K762E) and the PSKR1-KD(T998A) and PSKR1-KD(T998E) isoforms.

(A) Schematic indicating the phosphosite analyzed.

(B) The kinase isoforms (0.25 µg) were incubated with ^{32}P-ATP and the substrate MBP (0.5 µg). The autoradiograph (top) shows auto- and transphosphorylation activities. A Coomassie-stained gel (bottom) shows loading of PSKR1-KDs and the substrate MBP; M = size marker in kDa.

(C) ^{32}P incorporated in PSKR1-KDs and MBP was quantified by liquid scintillation counting. Results are averages (± SE) from three independent experiments with two technical replicates each. Significantly different values are indicated by different lower case (autophosphorylation) or capital (transphosphorylation) letters (Kruskal-Wallis, *p*<0.05).

Supplementary Figure S4

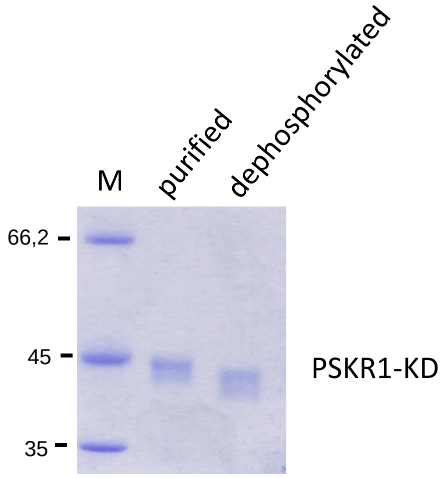

Supplementary Figure S4: Ectopically expressed, purified PSKR1-KD was treated with λ-phosphatase at room temperature for 2 h according to Muleya et al., (2016). The shift in mobility after dephosphorylation suggests that the protein is phosphorylated *in E.coli*.

REFERENCE

Muleya V, Marondedze C, Wheeler JI, Thomas L, Mok YF, Griffin MD, Manallack DT, Kwezi L, Lilley KS, Gehring C, Irving HR. 2016. Phosphorylation of the dimeric cytoplasmic domain of the phytosulfokine receptor, PSKR1. Biochem Journal **473**, 3081-98.

Supplementary Figure S5

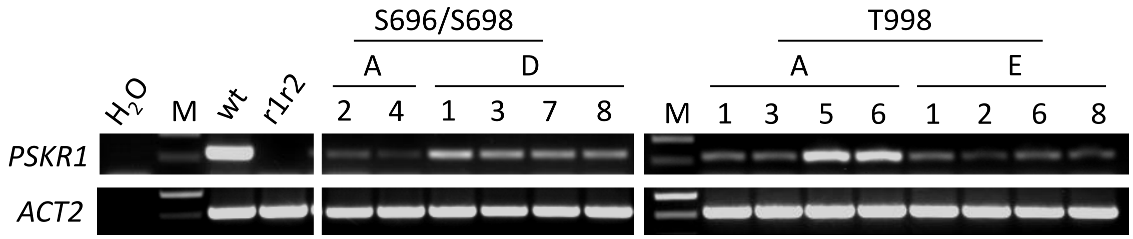

Supplementary Figure S5: *PSKR1* expression of PSKR1 isoforms in the *pskr1-3 pskr2-1* background was analyzed by reverse transcription PCR. Numbers indicate independent T-DNA-insertion lines. M = marker, wt = wild type, *r1r2* = *pskr1-3 pskr2-1*.
